# Supplementary material for: A low pre-infall mass for the Carina dwarf galaxy from disequilibrium modelling
Source: Nat Commun. 2015 Jul 2;6:7599. doi: 10.1038/ncomms8599 (PMC4506502; doi:10.1038/ncomms8599)
Supplement: Supplementary Information — Supplementary Figures 1-8 and Supplementary Tables 1-2 [file ncomms8599-s1.pdf]

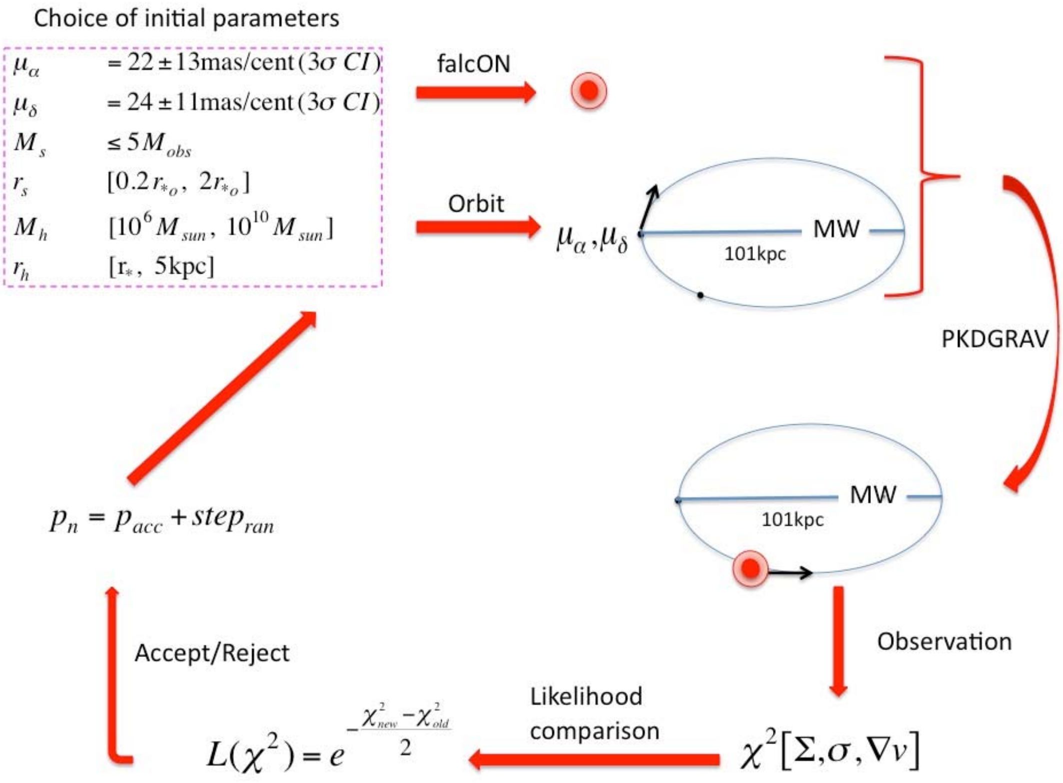

SUPPLEMENTARY FIGURE 1. Schematic representation of the algorithm and the codes. Starting from a random choice of values in the initial parameter space, the figure shows each step that the MCMC algorithm takes and the main codes used.

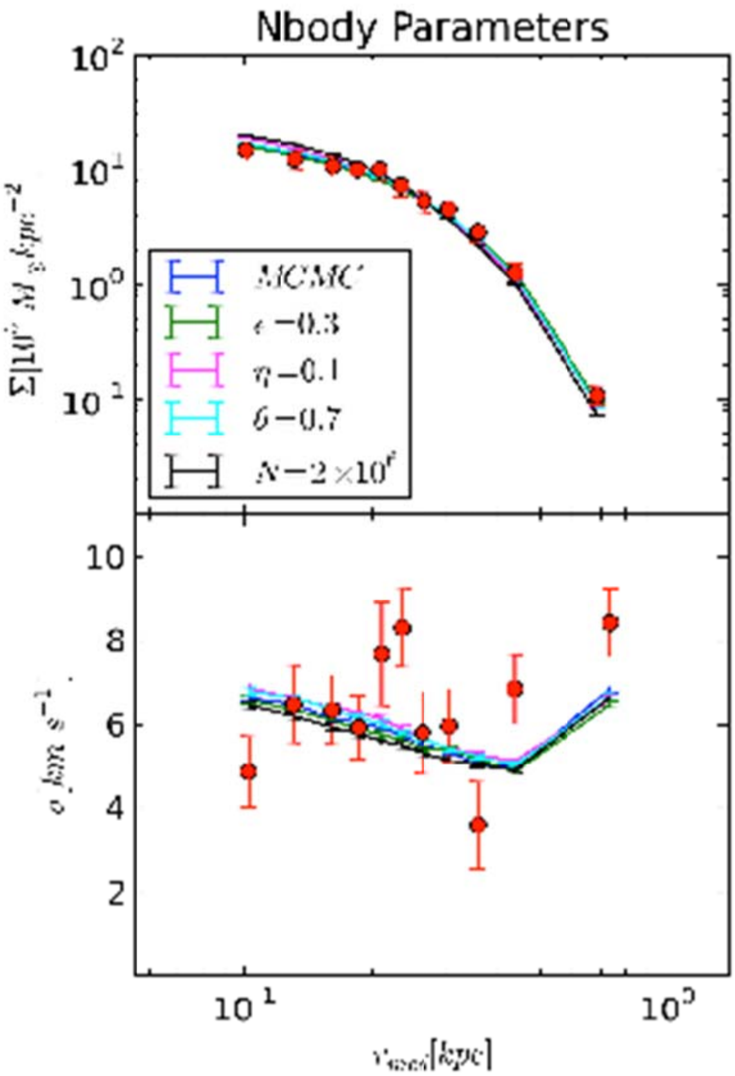

SUPPLEMENTARY FIGURE 2. Parameters of the Nbody simulations. Four simulations with different parameters used in the PkdGRAV Nbody code. The red points are the Carina data, while MCMC is the model run with the same parameters as those used in the MCMC chains (N=2 x 10^5, dη=0.1, θ=1, ε=50pc). Green, magenta, cyan and black are the models with different ε, dη, θ and N values given in the legends. The error bars are the Poissonian error calculated in each bin.

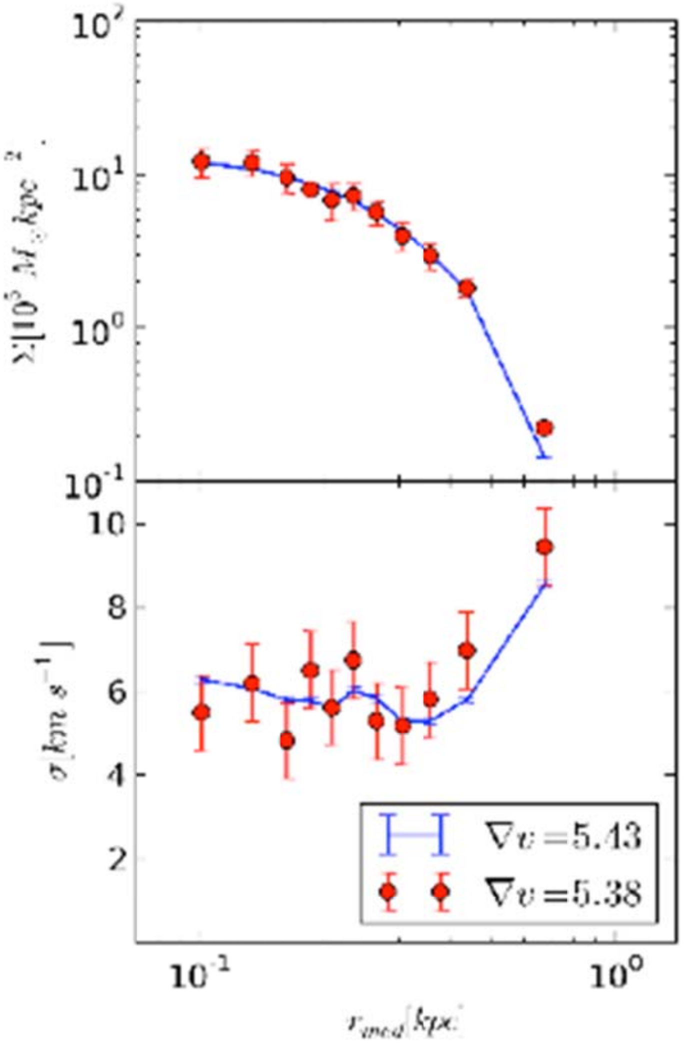

SUPPLEMENTARY FIGURE 3. Mock dwarf. The blue lines show the radial profiles for the surface brightness (top), and the velocity dispersion (bottom) for the Nbody model chosen as the Mock dwarf. The red points are obtained by adding noise to these as described in the text, and show the data we used as the "observations" in the MCMC chains of the Mock dwarf. . The error bars are the Poissonian error calculated in each bin.

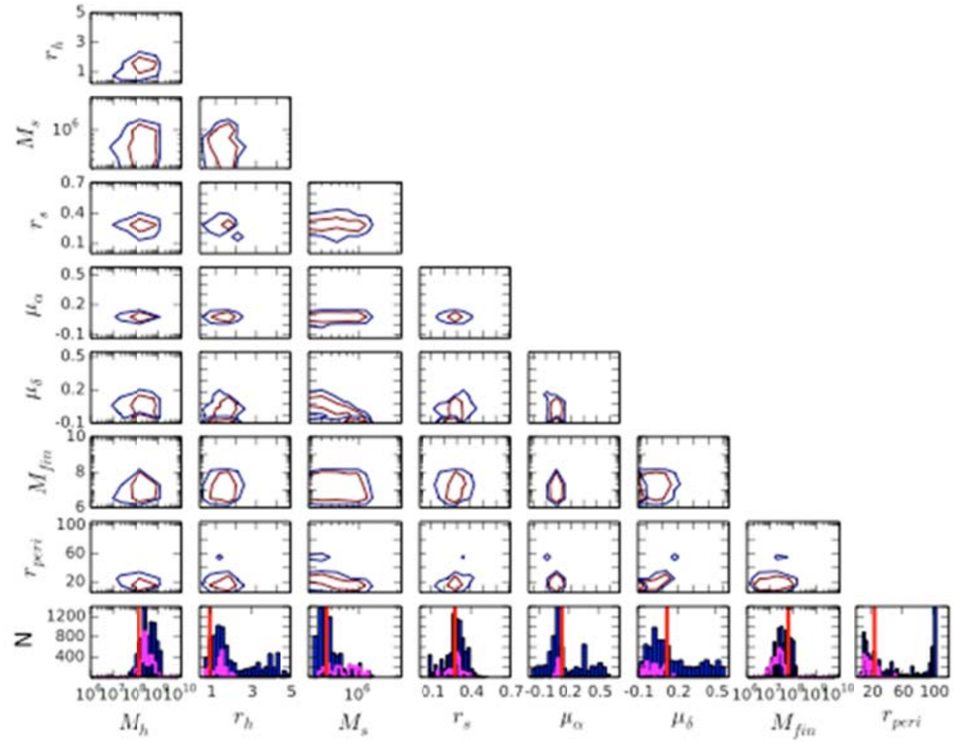

SUPPLEMENTARY FIGURE 4. Distribution of the models for the cusped MCMC chains for the Mock dwarf. The columns correspond to the 6 parameters of the initial parameter space (stellar mass and scale radius, total initial halo mass and scale radius, proper motions), the final mass within radius probed by the data and finally the perigalactic distance. The blue histograms on the bottom row show the distribution of all of the accepted models, while the magenta histograms include models with χ^2<9 only. The vertical red lines are the values that the chains try to recover.

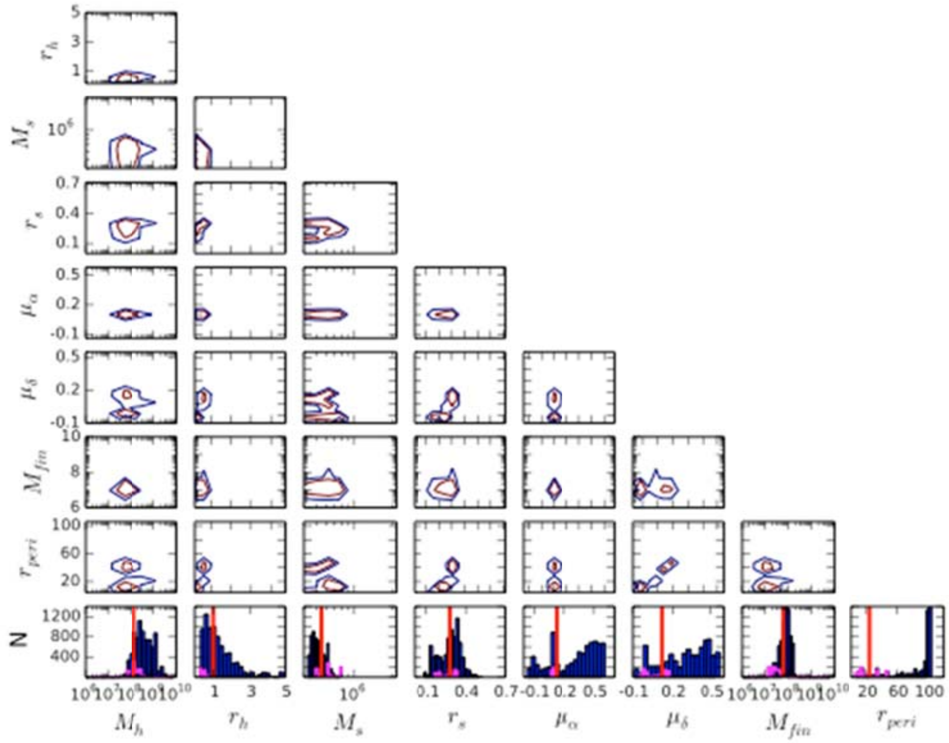

SUPPLEMENTARY FIGURE 5. Distribution of the models for the cored MCMC chains for the Mock dwarf. The columns and rows are the same as in Supplementary Figure 4 but for the cored chains.

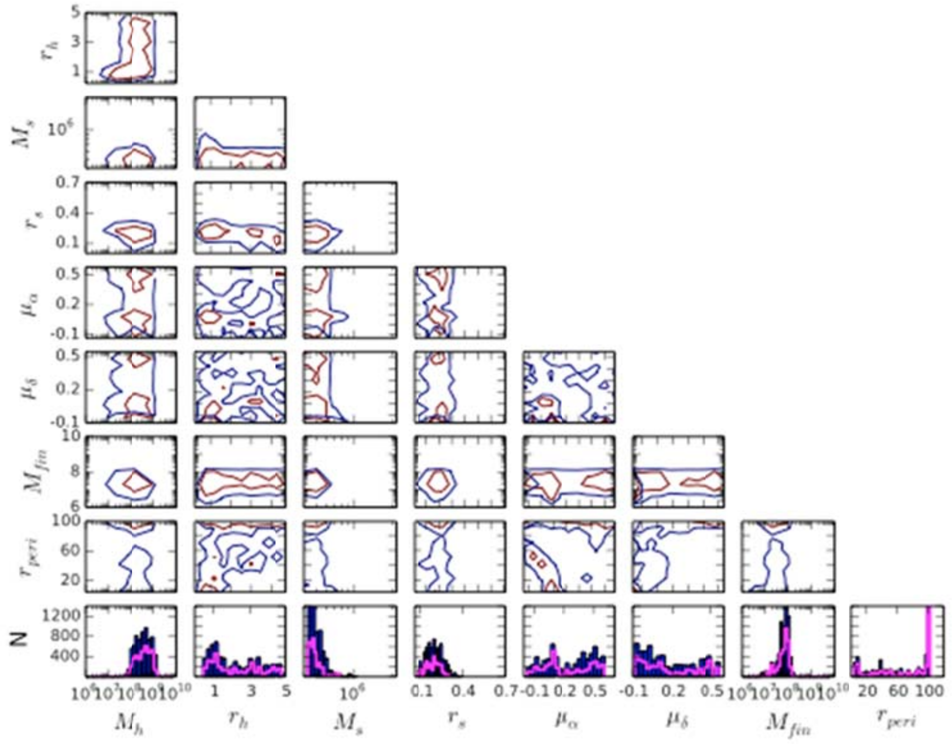

SUPPLEMENTARY FIGURE 6. Cusped MCMC chains for Carina with a χ^2<9 cut. The columns correspond to the 6 parameters if the initial parameter space (stellar mass and scale radius, total initial halo mass and scale radius, proper motions), the final mass within the radius probed by the data and finally the perigalactic distance. The blue histograms on the bottom row show the distribution of all accepted models, while the magenta histograms include models with χ^2<9 only.

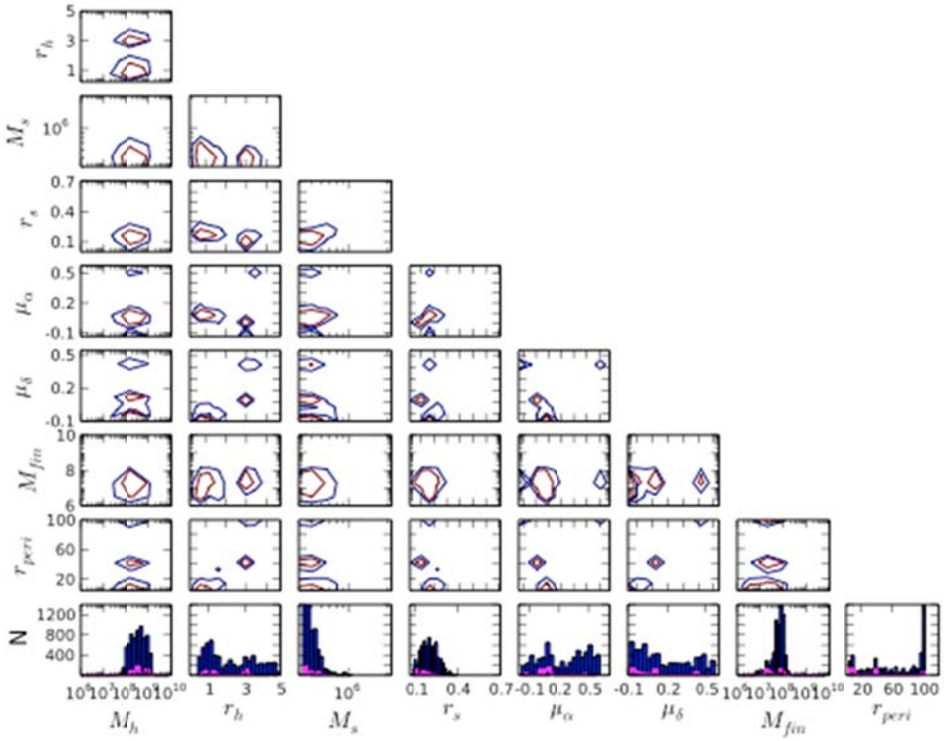

SUPPLEMENTARY FIGURE 7. Cusped MCMC chains for Carina with a χ^2<6 cut. The columns and rows are the same as in Supplementary Figure 6 for a χ^2<6.

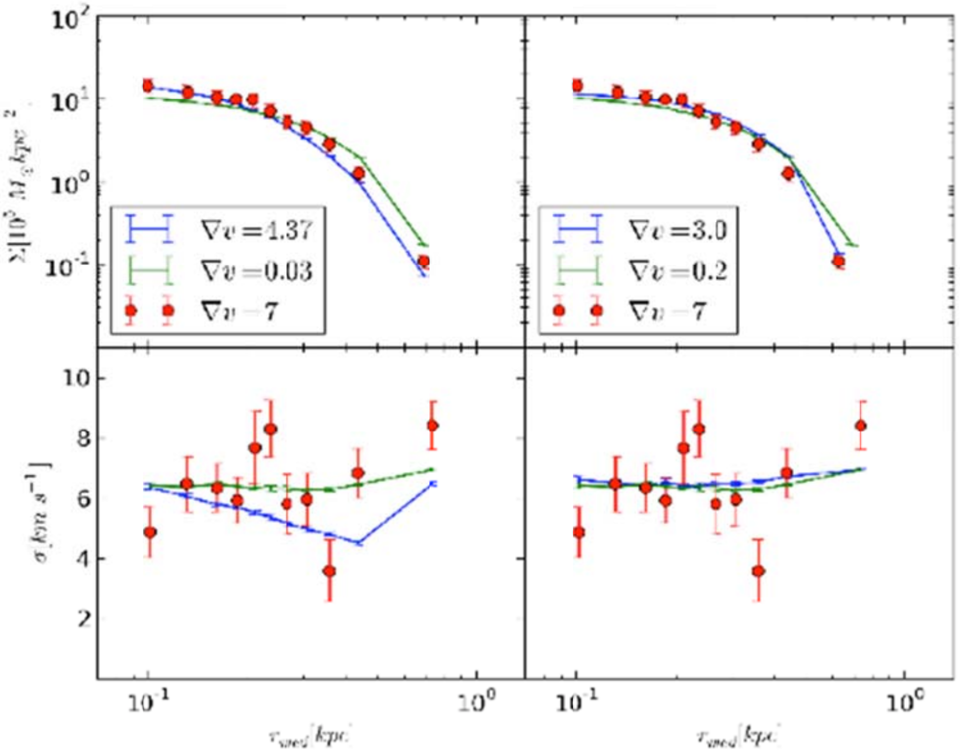

SUPPLEMENTARY FIGURE 8. Carina models with different χ^2. This figure is similar to Figure 3 where we plotted the best Carina models as well as the observational profiles. The velocity gradient ∇v is given in km s^{-1}. Here, we show models with larger χ^2 that we accepted in the cusped (a- on the left) and cored (b- on the right) chains. The red points are the observed profiles, while the blue lines are models with χ^2=5 and green are models with χ^2=9. . The error bars are the Poissonian error calculated in each bin.

N	ε (pc)	dη	θ	χ^2 (Σ,σ,∇v)
2 x 10^5	50	0.25	1.0	3.0
2 x 10^5	50	0.1	1.0	3.9
2 x 10^5	50	0.25	0.7	3.1
2 x 10^5	30	0.25	1.0	3.1
2 x 10^6	50	0.25	1.0	6.9

Supplementary Table 1. Results of the tests for different numerical parameters for PkdGRAV. The columns give the number of particles in the simulation (N), the softening parameter (ε), the fraction of the local dynamical time which sets the time

step ($d\eta$) and the reduced χ^2 between each simulation and the observed data as used in the MCMC.

	Original	CUSP ($\Sigma,\sigma,\nabla v$)	CORE ($\Sigma,\sigma,\nabla v$)	CUSP (Σ,σ)
M_s [10^5 Msun] (pre-infall)	5.5	7.9 (-2.4;+2.1)	6.2 (-1.2;+1.7)	5.1 (-0.5;+0.6)
r_s [kpc]	0.28	0.29 (-0.06;+0.05)	0.28 (-0.08;+0.05)	0.32 (-0.05;+0.05)
M_h [10^8 Msun]	1.4	2.79 (-0.62;+2.0)	1.42 (0.66;+0.86)	2.79 (-1.62;+3.52)
r_h [kpc]	1.0	1.44 (-0.44;+0.18)	0.37 (-0.05;+0.19)	2.2 (-1.06;+1.8)
$\mu_\alpha \cos(\delta)$ [mas cent^{-1}]	0.125	0.08 (-0.01;+0.02)	0.09 (-0.02;+0.19)	0.0 (-0.0;+0.49)
μ_δ [mas cent^{-1}]	0.12	0.02 (-0.06;+0.08)	0.0 (-0.02;+0.18)	0.0 (-0.0;+0.48)
R_{peri} [kpc]	24	12.6 (-4.6;+12.1)	7.7 (-0.2;+38.9)	100.8 (-35.1;+1.0)
M_{200} [10^8 Msun] (pre-infall)	1.34	2.69 (-0.6;+2.0)	1.37 (-0.6;+0.9)	2.66 (-1.5;+3.3)
M(r<1.5kpc) [10^7 Msun]	2.1	2.4 (-1.1;+0.8)	2.6 (-0.9;+1.3)	5.7 (-1.9;+2.5)
$v_{max,in}$(pre-infall) [km s^{-1}]	13.5	18.1 (-3.5;+3.9)	16.8 (-6.0;+4.3)	14.4 (-3.0;+3.3)
v_{max}(present) [km s^{-1}]	9.7	10.7 (- 2.3;+1.0)	11.2 (-1.7;+1.3)	13.3 (-2.4;+3.2)

Supplementary Table 2. PARAMETER CONSTRAINTS FOR MOCK DWARF.

The organisation is the same as Table 1. The first column lists all the parameters we included in our analysis, while column 2 shows the values of the parameter that the mock dwarf had. Columns three and four list the constraints we obtain for all parameters for chains that used cored or cusped models and included the velocity gradient in the model likelihood, while the fifth column presents the results for chains with cusped models in which the velocity gradient was ignored.
